# The Task-Relevant Attribute Representation Can Mediate the Simon Effect

**DOI:** 10.1371/journal.pone.0090954

**Published:** 2014-03-11

**Authors:** Dandan Tang, Xiao Zhao, Antao Chen

**Affiliations:** 1 Key Laboratory of Cognition and Personality (Ministry of Education) and School of Psychology, Southwest University, Chongqing, China; 2 School of Psychology, Liaoning Normal University, Dalian, Liaoning, China; University of Akron, United States of America

## Abstract

Researchers have previously suggested a working memory (WM) account of spatial codes, and based on this suggestion, the present study carries out three experiments to investigate how the task-relevant attribute representation (verbal or visual) in the typical Simon task affects the Simon effect. Experiment 1 compared the Simon effect between the *between-* and *within-category* color conditions, which required subjects to discriminate between red and blue stimuli (presumed to be represented by verbal WM codes because it was easy and fast to name the colors verbally) and to discriminate between two similar green stimuli (presumed to be represented by visual WM codes because it was hard and time-consuming to name the colors verbally), respectively. The results revealed a reliable Simon effect that only occurs in the *between*-*category* condition. Experiment 2 assessed the Simon effect by requiring subjects to discriminate between two different isosceles trapezoids (*within-category* shapes) and to discriminate isosceles trapezoid from rectangle (*between-category* shapes), and the results replicated and expanded the findings of Experiment 1. In Experiment 3, subjects were required to perform both tasks from Experiment 1. Wherein, in Experiment 3A, the *between-category* task preceded the *within-category* task; in Experiment 3B, the task order was opposite. The results showed the reliable Simon effect when subjects represented the task-relevant stimulus attributes by verbal WM encoding. In addition, the response times (RTs) distribution analysis for both the *between- and within-category* conditions of Experiments 3A and 3B showed decreased Simon effect with the RTs lengthened. Altogether, although the present results are consistent with the temporal coding account, we put forth that the Simon effect also depends on the verbal WM representation of task-relevant stimulus attribute.

## Instruction

In the visual Simon task, the stimulus simultaneously encompasses the spatial (task-irrelevant) and non-spatial (task-relevant) attributes. Subjects are typically required to respond to a left- or right-side presented stimulus according to the non-spatial attribute (e.g., colors or shapes). For example, if a red or green square is assigned to the left or right responses, respectively, the response will be faster and/or accuracy will be higher when the spatial location (left- or right-side) and the location of response to the stimuli are compatible (e.g., a red square is presented at the left) than when they are not (e.g., a red square is presented at the right). The Simon effect is indexed by the differences between the incompatible and the compatible conditions for the response times (RTs) and/or accuracy, which derives from that the task-irrelevant response automatically activated by the task-irrelevant spatial location interferes with the task-relevant response reflecting the stimulus-response (S-R) mapping instructions [1], [2], [3], [4].

However, it still remains unclear how the encoding of task-relevant stimulus attribute causes the Simon effect. The traditional accounts [e.g., the attention-shift account and the reference-coding account, see Stoffer [5] and Hommel [6], respectively] focus on how the stimulus attributes are encoded at the early processing stage. However, the dual route model [7] emphasizes that the competition between task-relevant response activation (non-spatial attribute) and task-irrelevant response activation (spatial attribute) at response selection stage is crucial to the generation of the Simon effect. That has been confirmed by functional magnetic resonance imaging (fMRI) studies [8], [9], [10], which found that a broad network of regions across brain were activated in the Simon task and validated that the Simon effect was related to response selection. Subsequently, the response-discrimination account [11], [12] proposes that the Simon effect is generated when the spatial stimulus and response codes are represented and interact with each other in working memory (WM), but is absent without the WM representation.

A common view that the Simon effect is connected with some kind of manipulation in WM can converge from the later accounts of Simon effect, such as the dual-route model or the response-discrimination account. This view has been verified by Zhao, Chen, and West [13], who used a dual-task paradigm to explore whether the Simon effect was modulated by a concurrent WM load, and dramatically found that the Simon effect was abolished by the concurrent verbal WM load, but was not affected by the concurrent spatial WM load. Afterwards, Wühr and Biebl [14] used a similar paradigm and found that both visual and verbal WM load influenced the Simon effect. Thus, the types of WM codes used for representing the task-relevant stimulus might be critical for the generation of Simon effect.

Although some theories have proposed insightful explanations on the mechanism of occurrence of the Simon effect [15], [16], it is not clear what roles verbal and visual WM respectively play in the generation of the Simon effect. Based on Baddley's influential WM model [17], the WM system, which retains and manipulates information temporarily, is composed of three main components: the central executive system that controls and regulates the cognitive process, and two storage systems (the phonological loop that deals with language processing such as color naming and the visuospatial sketchpad that handles visuospatial information processing such as color or shade discrimination). Recently, such a model has been corroborated by Ikeda and Osaka [18], who used a 2-back paradigm to examine how *between- and within-category* colors were represented in WM. The results showed that *between- and within-category* colors were represented by different WM codes depending on what category they belong to. That was, the representation of *between-category* colors (e.g., red or blue, being easy to be named) strongly activated the verbal WM regions (the left inferior frontal gyrus and left inferior parietal lobule) corresponding to the phonological loop; whereas the representation of *within-category* colors (e.g., red 1 or red 2, being hardly discriminated by naming) strongly activated the visual WM region (the right inferior frontal gyrus) corresponding to the visuospatial sketchpad. Therefore, the results indicate that the processes of *between-* and *within-category* colors involve separated brain regions, which is accordant with the result of Rothmayr et al. [19] that showed hemispheric dissociation in representing information verbally and non-verbally. In addition, single-unit recording study with macaque monkeys had shown that many color-selective neurons existed within the inferior temporal (IT) cortex [20], which was strongly activated by color stimuli in imaging studies with monkeys [21], [22]. Thus, the IT cortex plays an important role in color vision [23].

Previously, Lammertyn, Notebaert, Gevers, & Fias in their Experiment 2 [24] used the colors with adjacent color difference as the task-relevant stimulus attribute to explore the mechanism of the Simon effect and found that the Simon effect decreased with the narrowed color distance. This verifies that (1) verbal WM encoding of the task-relevant stimulus attribute plays a critical role in the generation of the Simon effects and (2) a conversion from representing colors in verbal WM to representing colors in visual WM may occur because of the increasing difficulty of representing colors in verbal WM when color distance difference is lessened. Moreover, in Experiment 4, they revealed a diminished Simon effect when shapes (being hard to be named) were used as the task-relevant attribute [24]. In this case, subjects might adopt the visual WM coding strategy to represent the task-relevant stimulus attribute, which weakened the task-irrelevant spatial representation of stimulus and the corresponding response activation, and therefore reduced the Simon effect. Therefore, based on the fact that the task-relevant attribute in the Simon task was usually *between-category* colors or shapes (e.g., red and green; square and circle), which had been confirmed to be represented in verbal WM, and researches adopting such stimuli observed the reliable Simon effect [25], [26], [27], we assume that verbal WM encoding of the *between-category* colors or shapes causes the Simon effect because verbal WM representation and spatial dimension representation of the stimulus attributes are independent and do not interfere with each other; in this case, the task-irrelevant response activation can interfere with the task-relevant one. However, if the *within-category* colors or shapes are used as the task-relevant Simon stimuli, the visual WM coding strategy will be adopted to represent the stimulus attribute. Accordant with Experiment 4 of Lammertyn et al [24], we forecast the Simon effect will be eliminated. The present study tested the assumptions in the following three experiments.

Concretely, adopting a mixed factor design, Experiment 1 compared the Simon effect in a task which required discrimination between a red and a blue stimulus (*between-category* color condition), with the Simon effect in a task which required discrimination between two green stimuli (*within-category* color condition). Subjects were divided into two groups randomly. Group one performed the Simon task with the *between-category* color patches used as the task-relevant Simon stimuli. Group two performed the same task with the *within-category* color patches used as the task-relevant Simon stimuli. All subjects were instructed just to differentiate the color of stimuli and make an assigned response based on the color of patch. If the verbal WM coding strategy was adopted to represent the task-relevant stimulus colors, we expected a significant Simon effect will occur in group one but not in group two.

Experiment 2 (a mixed factor design), assessed the Simon effect in tasks, which required subjects to discriminate between two types of isosceles trapezoids with different basic angles and to discriminate between isosceles trapezoid and rectangle. In the former task, the stimuli were hardly discriminated by naming (*within-category* condition); in the later task, the stimuli were discriminated easily by naming the shapes (*between-category* condition). As the only difference between Experiments 1 and 2 was the stimulus formats (colors in Experiment 1; shapes in Experiment 2), we expected the results of Experiment 2 would be identical to those of Experiment 1. If the results were confirmed, we would conclude that the Simon effect more likely relied on the verbal WM codes and was independent on the format of stimuli.

Specifically, the response-discrimination hypothesis claimed that the Simon effect resulted from the WM representation, which allows for the set effects [11]. That is, if a particular WM code is selected to represent a response in a task, the representation pattern may carry over to the subsequent task. Given this, Experiment 3 contained two parts; each adopted a within-subjects design and manipulated the task order to explore whether a special WM representation can be acquired from the previous task. Subjects performed both tasks from Experiment 1, with the *between-category* color condition preceding the *within-category* color condition in Experiment 3A, but with the task order reversed in Experiment 3B. If Experiments 3A and 3B show distinctly different patterns of the Simon effects for the *within-category* tasks, the set effect will be confirmed to depend on the task order.

## Experiment 1

In this experiment, we adopted a mixed factor design to explore the influence of color categories (*between-category*, *within-category*) on the Simon effect. If the Simon effect only occurred in the *between-category* color task, the prediction that the Simon effect relied on the verbal representation by the phonological loop could be verified.

### Method

#### Subjects

Fifty-two healthy undergraduates (24 males, mean age = 21.0 years old, *SD* = 3.3), took part in Experiment 1. All subjects had normal or corrected-to-normal vision, normal color perception, and were unconscious to the purposes of this experiment. Subjects were paid for their participation. All subjects gave written informed consent, and the local ethics committee of Southwest University (Chongqing, China) approved the experimental procedures, which were in accordance with the standards of the Declaration of Helsinki.

#### Apparatus and Stimuli

The experiment was conducted in a dimly lit room and run on a Dell PC which was controlled by the Eprime 1.1 software (Psychology Software Tools, Pittsburgh, PA). All stimuli were presented on a 17-inch TCL monitor with an 85-Hz refresh rate. And the computer screen was gray. Subjects sat approximately 65 cm away from the screen and responded by pressing one of two keys on a standard QWERTY keyboard.

The stimuli for both Simon tasks were two colored squares, with each subtending a visual angle of 0.71°×0.71°. These stimuli appeared 3.0° from the left or right of a centered white fixation with a visual angle of 0.3°×0.3°. The stimulus colors were red (RGB 255, 0, 0) and blue (RGB 0, 0, 255) in the *between-category* task and were green (RGB 29, 136, 56; RGB 80, 132, 0) in the *within-category* task. Averaged color differences (ΔEuv*) were 269.5 and 20.1 for the *between*- and *within-category* colors, respectively. For the compatible condition, the location of the stimulus and the assigned response key were on the same side. For the incompatible condition, they were on the opposite sides.

#### Design

Experiment 1 was a 2 (between-subjects factor, color category: *between-category*, *within-category*)×2 (within-subjects factor, compatibility: compatible, incompatible) mixed factors design. In the *between-category* task, the task-relevant stimuli consisted of *between-category* colors; in the *within-category* task, the task-relevant stimuli consisted of *within-category* colors. Subjects were randomly assigned to two groups (each group contained twenty-six subjects). Each of the Simon task included a practice block of 32 trials and an experimental block of 144 trials, sequentially.

#### Procedure

The procedure was the same for the *between*- and *within-category* tasks. Firstly, a 300-msec fixation point was presented at the central of screen, which was followed by a blank duration for 300–500 msec with a random interval. After the blank disappeared, a color patch was presented at the left or the right side of the center fixation point. It lasted until a response was given or disappeared after 1,500 msec without a response made.

Subjects were required to identify the color while ignoring the location of patch. In the *between-category* task, half of the subjects were instructed to press the “Q” key for the red patch with the left index finger and the “O” key for the blue patch with the right index finger. In the *within-category* task, half of the subjects were instructed to press the “Q” key for the light green patch with the left index finger and the “O” key for the dark green patch with the right index finger. The other half was vice versa. Subjects were instructed to perform the task as fast as possible without sacrificing accuracy.

### Results

**Figure 1:**
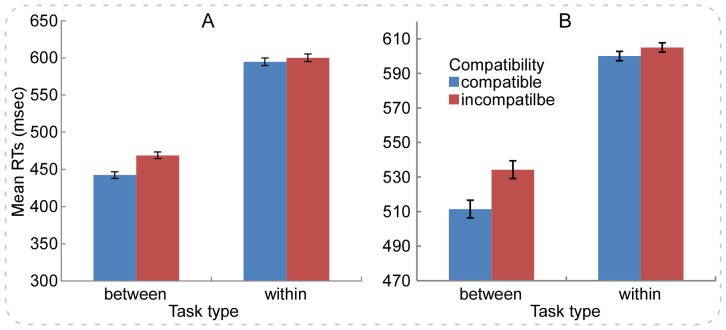
The RTs results for the *between-* and *within-category* Simon tasks in Experiments 1 and 2. Panel **A** illustrates that the Simon effect (incompatible minus compatible) is a function of task type (*between-* and *within-category* color tasks) in Experiment 1. Notably, the Simon effect disappears in the *within-category* task but is observed in the *between-category* task. Panel **B** illustrates that the Simon effect is a function of task type (*between-* and *within-category* shape tasks) in Experiment 2. As can be seen, the RTs patterns are extremely similar to those found in Experiment 1, i.e., the Simon effect disappears in the *within-category* task but is observed in the *between-category* task. Error bars indicate ±1 standard errors of the mean (*SEM*s) as calculated by the pairwise difference method (*SEM^pairedDiff^*) [39].

For the RTs analyses, the incorrect trials, and trials that the RTs were shorter than 150 msec or longer than 1,500 msec were eliminated. These criteria would also be adopted in the following Experiments. For the present experiment, we eliminated 5.4% and 9.3% of all trials for *between- and within-category* tasks, respectively. Fig. 1A showed the RTs data in Experiment 1, which revealed that the compatibility (compatible, incompatible) varied as a function of color category (*between-* and *within-category*). As depicted, the Simon effect (incompatible minus compatible) was smaller in the *within-category* condition than that in the *between-category* condition. Then, a mixed analysis of variance (ANOVA) was respectively performed for the RTs and error rates with color category (*between-* and *within-category*) as between-subjects variable and compatibility (compatible, incompatible) as within-subjects variable.

For the RTs, the mixed ANOVA revealed significant main effects of color category (142 msec), *F*(1,50) = 81.67, *p*<.001, *η* ^2^ = .62, and of compatibility (16 msec), *F*(1,50) = 22.19, *p*<.001, *η* ^2^ = .31. Importantly, the interaction between the two factors was significant, *F*(1,50) = 9.96, *p*<.01, *η* ^2^ = .17. Post hoc test revealed that the RTs (1) were longer for both the compatible and the incompatible conditions in the *within-category* relative to *between-category* task (*p*s<.001); (2) were longer for the incompatible relative to compatible condition in the *between-category* task (*p*<.001); and (3) were comparable between the compatible and the incompatible conditions in the *within-category* task (*p*>.3). These findings suggested that the color category had a robust modulatory effect on the size of Simon effect.

For the error rates, the mixed ANOVA revealed significant main effects of color category, *F*(1,50) = 5.20, *p*<.05, *η* ^2^ = .09, and of compatibility, *F*(1,50) = 4.33, *p*<.05, *η* ^2^ = .08. However, the interaction between the two factors was significant, *F*<1. The error rates were 4.1% and 4.3% for the compatible and incompatible trial types in the *within-category* task, respectively; and were 2.2% and 2.9% for the compatible and incompatible trial types in the *between-category* task, respectively. Thus, for the error rates, color category did not significantly mediate the size of Simon effect.

### Discussion

Accordant with the assumption, the results provided crucial evidence for the absence of Simon effect in the *within-category* task and the occurrence of it in the *between-category* task, which were likely attributed to the manipulations of stimulus format that resulted in different representations of stimulus attribute in WM. The Simon effect profited from the verbal WM representation of *between-category* colors but was eliminated by visual WM representation of those, which was also consistent with the previous findings revealing that when stimuli or responses were verbal, performance was mediated in part by activation of a verbal-name code for the Simon stimulus [16].

It should be noted that most of Simon task used the colors or shapes as the task-relevant stimulus attribute, and the obtained results accordantly showed significant Simon effect. Accordingly, we adopted a shape-category Simon task in Experiment 2 to replicate and expand the present results.

## Experiment 2

The aim of Experiment 2 was to expand the findings of Experiment 1 to the Simon task adopting geometric shapes as the task-relevant attribute. To this end, Experiment 2 used a 2 (between-subjects factor, shape category: *between-category*, *within-category*)×2 (within-subjects factor, compatibility: compatible, incompatible) mixed factor design, which was similar to that used in Experiment 1 except that the color-category stimuli were replaced by the shape-category stimuli. That was, in the *between-category* task, the stimuli consisted of the *between-category* shapes (one black isosceles trapezoid and one black rectangle, the areas of which were equal); in the *within-category* task, the stimuli consisted of the *within-category* shapes (two black isosceles trapezoids with equal areas but different degrees of base angles). We predicted that the results would be similar to those of Experiment 1, i.e., in the *within-category* task, the Simon effect would disappear (as the shapes had identical geometric name, subjects had to discriminate and characterize them visually); by contrast, in the *between-category* task, the effect was still robust, which was accordant with most of previous studies [28], [29], [30].

### Method

#### Subjects

Sixty-one right-handed healthy undergraduates (27 males, mean age = 20.8 years old, *SD* = 1.5) were recruited and randomly assigned to two groups, with group one (thirty-four subjects) performing the *between-category* task and group two (twenty-seven subjects) performing the *within-category* task. All subjects had normal or corrected-to-normal vision, normal color perception, and were unconscious to the purposes of this experiment. They were paid for their participation. All subjects gave written informed consent, and the local ethics committee of Southwest University (Chongqing, China) approved the experimental procedures, which were in accordance with the standards of the Declaration of Helsinki.

#### Stimuli

The stimuli in the *within-category* task were two black isosceles trapezoids, which were similar except the difference in the degrees of base angles. The stimuli in the *between-category* task were one black isosceles trapezoid and one black rectangle. The areas of all shapes were equal. In addition, all stimuli subtended a visual angle of less than 1.42°×0.71° and appeared 3.0° from the left or right of a central fixation with a visual angle of 0.3°×0.3°.

#### Apparatus, Procedure, and Design

These were exactly the same as those used in Experiment 1. In addition, subjects were required to make a response according to the shape of stimuli. In the *within-category* task, half of the subjects were instructed to press the “Q” key with the left index finger and the “O” key with the right index finger for the two types of isosceles trapezoid, respectively. The other half was vice versa. In the *between-category* task, half of the subjects were instructed to press the “Q” key with the left index finger and the “O” key with the right index finger for the black isosceles trapezoid and the black rectangle, respectively. The other half was vice versa.

### Results

For the analysis of the RTs, we excluded 9.2% and 9.3% of all trials for *between- and within-category* tasks, respectively. Fig. 1B showed the RTs data in Experiment 2, revealing that the compatibility (compatible, incompatible) varied as a function of shape category (*between-* and *within-category*). As depicted in Fig. 1B, the Simon effect (incompatible minus compatible) was smaller in the *within-category* condition than that in the *between-category* condition. Similar to Experiment 1, the mixed ANOVA was firstly performed for the RTs with shape category (*between-* and *within-category*) as between-subjects variable and compatibility (compatible, incompatible) as within-subjects variable. The results revealed significant main effects of shape category (80 msec), *F*(1,59) = 31.23, *p*<.001, *η* ^2^ = .35, and of compatibility (14 msec), *F*(1,59) = 25, *p*<.001, *η* ^2^ = .30, respectively. Importantly, a significant interaction between the two factor was found, *F*(1,59) = 10.96, *p*<.002, *η* ^2^ = .16. Post hoc test revealed that the RTs (1) were longer for both the compatible and the incompatible conditions in the *within-category* relative to *between-category* task (*p*s<.001); (2) were longer for the incompatible relative to compatible condition in the *between-category* task (*p*<.001); and (3) were comparable between the compatible and the incompatible conditions in the *within-category* task (*p* = .2). The results implied that the Simon effect disappeared in the *within-category* task; however, the Simon effect appeared in the *between-category* task (Fig. 1B). Note that the findings of Experiment 2 were extremely similar to those of Experiment 1, which indicated that Experiment 2 expanded the findings of Experiment 1 by adopting the shape as task-relevant attribute in the Simon task.

For the error rates, the mixed ANOVA revealed that there were no any significant effects found, *ps*>.19, indicating that shape category did not significantly mediate the size of Simon effect. The error rates were 4.4% and 4.6% for the compatible and incompatible trial types in the *within-category* task, respectively; and were 3.9% and 5.1% for the compatible and incompatible trial types in the *between-category* task, respectively.

### Discussion

Experiment 2, using the shape-category Simon task, confirmed and expanded the findings of Experiment 1. The results were also consistent with our assumption. When the *within-category* shapes (two types of isosceles trapezoids with different degrees of base angles) were used as the task-relevant stimulus attribute that had to be represented by visual WM, the Simon effect disappeared; when the *between-category* shapes (rectangle and isosceles trapezoid) were used as the task-relevant stimulus attribute that could be represented by verbal WM, the effect appeared. Thus, we concluded that the generation of Simon effect depended on the verbal WM representation of the task-relevant attribute but not on a particular stimulus modality.

## Experiment 3

Experiment 3 consisted of two parts. Experiment 3A adopted a within-subjects design, where all subjects performed both tasks from Experiment 1, with the *between-category* condition preceding the *within-category* condition. The aim was to explore whether the verbal representation in the former task could be carried over to the later task (set effect). In the *within-category* task, if subjects learned to represent the *within-category* color by the verbal WM encoding, a reliable Simon effect would be observed; on the contrary, the Simon effect would disappear. Experiment 3B adopted the same design as that used in Experiment 3A; however, the *within-category* condition was prior to the *between-category* condition. Such a manipulation would provide an appropriate comparison with Experiment 3A. If verbal encoding was a critical factor, the Simon effect should be absent in the *within-category* condition because the task-relevant stimulus attribution still was represented by the visual WM code without previous experience; but it should be present in the following *between-category* condition because the task-relevant stimulus attribution could be represented by the verbal WM code. If the prospections of both experiments were validated, our conclusion that verbal WM representation was critical in inducing the Simon effect would be further supported.

### Experiment 3A

#### Subjects

Thirty-five right-handed undergraduates (13 males, mean age = 21.5 years old, *SD* = 1.4), took part in Experiment 3A. All subjects had normal or corrected-to-normal vision, normal color perception, and were unconscious to the purposes of this experiment. They were paid for their participation. All subjects gave written informed consent, and the local ethics committee of Southwest University (Chongqing, China) approved the experimental procedures, which were in accordance with the standards of the Declaration of Helsinki.

#### Apparatus, Stimuli, Procedure, and Design

These were exactly the same as those used in Experiment 1, except that Experiment 3A adopted a 2 (color category: *within-* and *between-category* color)×2 (congruency: compatible, incompatible) within-subjects design. Note that subjects performed the *between-category* task preceding the *within-category* task.

#### Results

**Figure 2:**
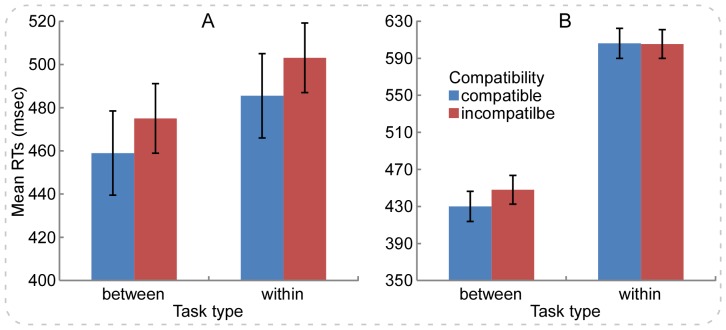
The RTs results for Experiment 3. Panel **A** illustrates the RTs results for Experiment 3A with the *between-category* color task preceding the *within-category* color task. It is notable that the Simon effect is clearly observed and the RTs levels are similar in both tasks. Panel **B** illustrates the RTs results of Experiment 3B with the *with-category* color task preceding the *between-category* color task. Similar to Experiments 1 and 2, Experiment 3B reveals significant Simon effect in the *between-category* but not *within-category* tasks. Error bars indicate ±1 *SEM*s as calculated by the pairwise difference method (*SEM^pairedDiff^*) [39].

For the analysis of the RTs, 7.4% and 9.1% of all trials for *between- and within-category* tasks were eliminated, respectively. Fig. 2A showed the RTs data in Experiment 3A, revealing that the Simon effect (incompatible minus compatible) occurred in both *between-* and *within-category* conditions. A two-way repeated-measures ANOVA was performed for the RTs with the color category (*within-* and *between-category* color) and compatibility (compatible, incompatible) as within-subjects variables. The results revealed a significant main effect of compatibility (17 msec), *F*(1,34) = 26.31, *p*<.001, *η* ^2^ = .44; however, neither the main effect of color category nor the interaction between the two factor were significant, *p*s>.1. The non-significant interaction implied that the color category did not influence the size of Simon effect. Then, the separate paired-sampled *t* test (2-tailed) revealed significant difference between the incompatible and the compatible trial types for both the *within-* and *between-category* tasks, RT difference = 18 msec, *t*(34) = 3.93, *p*<.001, and RT difference = 16 msec, *t*(34) = 3.25, *p*<.01, respectively. These findings suggested that the size of the Simon effect was significant in both tasks (Fig. 2B).

For the error rates, the two-way repeated-measures ANOVA was run with color category and compatibility as within-subjects variables. However, there were no significant effects obtained, *p*s>.06. The error rates were 4.4% for both the compatible and the incompatible trial types in the *within-category* task; and were 2.9% and 3.9% for the compatible and incompatible trial types in the *between-category* task, respectively.

### Experiment 3B

#### Subjects

Thirty-four right-handed undergraduates (11 males, mean age = 21.21 years old, *SD* = 1.20), took part in Experiment 3B. All subjects had normal or corrected-to-normal vision, normal color perception, and were unconscious to the purposes of this experiment. They were paid for their participation. All subjects gave written informed consent, and the local ethics committee of Southwest University (Chongqing, China) approved the experimental procedures, which were in accordance with the standards of the Declaration of Helsinki.

#### Apparatus, Stimuli, Procedure, and Design

These were exactly the same as those used in Experiment 3A, except that subjects performed the two tasks in the opposite order, i.e., subjects performed the *within-category* task preceding the *between-category* task.

#### Results

For the analysis of the RTs, 11.6% and 6.3% of all trials for *between- and within-category* tasks were eliminated, respectively. Fig. 2B showed the RTs data in Experiment 3B, revealing that the Simon effect (incompatible minus compatible) occurred only in the *between-category* task. The two-way repeated-measures ANOVA was performed for the RTs with the color category (*within-* and *between-category* color) and the compatibility (compatible, incompatible) as within-subjects variables. The results revealed a non-significant main effect of compatibility (9 msec), *F*(1,33) = 3.85, *p*>.05, *η* ^2^ = .10; however, the main effect of color category was significant (167 msec), *F*(1,33) = 114.35, *p*<.001, *η* ^2^ = .78; the interaction between the two factor was significant, *F*(1,33) = 8.88, *p*<.005, *η* ^2^ = .21. Post hoc test revealed that the RTs (1) were longer for both the compatible and the incompatible conditions in the *within-category* relative to *between-category* task (*p*s<.001); (2) were longer for the incompatible relative to compatible condition in the *between-category* task (*p*<.001); and (3) were comparable between the compatible and the incompatible conditions the *within-category* task (*p* = .9). These findings suggested that the color category robustly modulated the size of Simon effect (Fig. 2B).

For the error rates, the two-way repeated-measures ANOVA was run with color category and compatibility as within-subjects variables. The results revealed a non-significant main effect for the compatibility, *F*(1,33)<1; however, the main effect of color category was significant, *F*(1,33) = 13.32, *p*<.001, *η* ^2^ = .29; the interaction between the two factor was significant, *F*(1,33) = 4.45, *p*<.05, *η* ^2^ = .12. Post hoc test revealed that for the compatible trials, the error rates were significantly higher in the *within-category* task than those in the *between-category* task, *p*<.005. The error rates were 6.7% and 4.7% for the compatible and incompatible trial types in the *within-category* task, respectively; and were 2.5% and 3.4% for the compatible and incompatible trial types in the *between-category* task, respectively.

### Discussion of Experiments 3

In Experiment 3, subjects performed both tasks from Experiment 1 in a fixed order, with the *between-color* condition preceding the *within-color* condition in Experiment 3A, but a reversed order in Experiment 3B. As a result, Experiment 3A revealed the reliable Simon effect in both *between-* and *within-category* tasks with quite similar RTs levels (Fig. 2A). Compared with the findings of Experiment 1 (the reliable Simon effect only occurred in the *between-category* task), Experiment 3A confirmed our predictions that the verbal WM representation could contribute to the occurrence of the Simon effect and could be transformed from the *between-category* task to the *within-category* task, i.e., subjects could learn to use the verbal WM to represent the *within-category* color. Therefore, the results provided evidence for that the occurrence of the Simon effect depended on the verbal WM representation.

Experiment 3B revealed the reliable Simon effect only in the *between-category* task (Fig. 2B). That further indicated that subjects might adopt a visual WM representation to process the task-relevant stimulus attribution in the *within-category* task because the color could not be named verbally. In this case, visual WM representation weakened representation of the spatial attribute of the stimulus, resulting in that the task-irrelevant response activation did not interfere with the task-relevant response in the incompatible trial type, and therefore the Simon effect disappeared. In addition, the reliable Simon effect was observed in the following *between-category* task, which indicated that the visual WM encoding did not be used to represent the *between-category* colors as they were easily named in the first place. Therefore, the findings of Experiment 3 supported for our conclusion that the verbal WM representation was critical in inducing the Simon effect.

As implied by Figs. 1 and 2, the overall RTs levels were longer in the *within-category* tasks than those in the *between-category* tasks. The lengthened RTs might result in the decreased Simon effect in the *within-category* tasks. Many previous studies validated that the Simon effect decreased or even dissolved with lengthened RTs [6], [26], [31], [32], [33], which was typically attributed to rapid activation of the task-irrelevant response code (the spatial location of stimulus) and then prompt decrease of that activation (for a review, see [34]).

**Figure 3:**
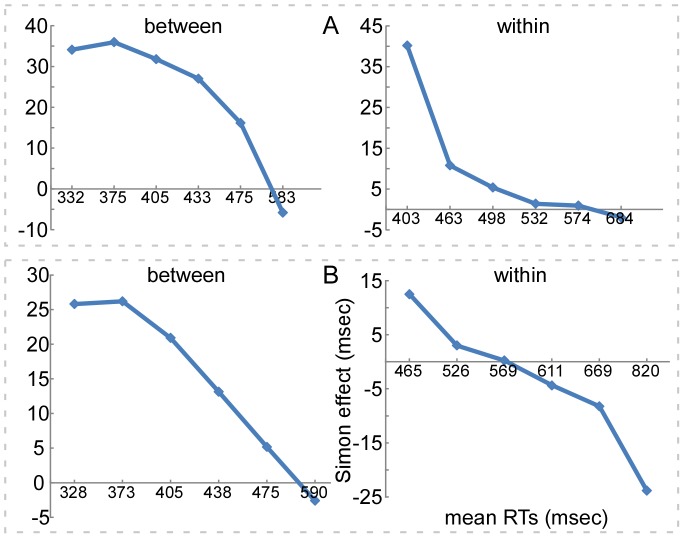
The time course of the Simon effect in Experiment 3. X-axis, mean RTs (msec), divided into six quantiles; y-axis, the Simon effect (msec), calculated by subtracting compatible from incompatible trial type. Panel **A** illustrates the time course of the Simon effect for Experiment 3A (the *between-category* color task preceding the *within-category* color task). It is notable that although the effect typically yields a monotonic decreasing function with increased RTs in both tasks, the slopes are distinctly different in both tasks. Panel **B** illustrates the time course of the Simon effect for Experiment 3B (with the task order reversed). Left: The Simon effect keeps stable at the first two bins, but almost reduces linearly at the last four bins. Right: The Simon occurs in the first two bins, whereas disappears at the third bin, even reverses by the last three bins.

To estimate the time course of the Simon effect, we respectively conducted the RTs distribution analysis [26], [34] for Experiments 3A and 3B, where the mean RTs were divided into six quantiles. Figs. 3A and 3B respectively depicted the time course of the Simon effect (maintaining about 100 msec), which was a decreasing function of mean RTs for each bin, in Experiments 3A and 3B. Although the patterns were seemingly consistent with the temporal activation account [26], [35], we argued that the verbal WM representation of task-relevant attribute was potential fundamental for the generation of the Simon effect because it reflected a flexible and efficient cognitive processing pattern that could be acquired according to both previous experience and current task requires.

## General Discussion

The present study used the Simon task to investigate the relationship between the Simon effect (incompatible minus compatible) and the representation of the task-relevant stimulus attribute in WM. Experiments 1 and 2 indicated that the Simon effect occurred when the task-relevant attributes (e.g., the *between-category* colors or shapes) were easy to be discriminated by naming and were represented in the verbal WM by phonological loop, but disappeared when the task-relevant attributes (e.g., *within-category* colors or shapes) were hardly discriminated and were represented by the visual WM encoding. The findings were consistent with the previous studies [28], [29], [30]. Experiment 3, adopting the same tasks as those used in Experiment 1 and manipulating the task order, investigated the set effect. Experiment 3A found the significant Simon effect in the *within- and between-category* tasks. Experiment 3B only obtained the significant Simon effect in the *between-category* task. Moreover, in Experiment 3, the time course of the Simon effect was revealed by the RTs distribution analysis, which implied that although the Simon effect decreased with the lengthened RTs, the slopes of the function were distinctly different between the *between- and within-category* tasks. In summary, the present study demonstrates that (1) the verbal WM representation is crucial for the generation of Simon effect; (2) the Simon effect is independent on the used task-relevant stimulus attribution; and (3) the set effect depends on the task order (the verbal WM representation can be transformed from the *between-category* to the *within-category* color task).

In Experiment 1, the only difference between the *within- and between-category* tasks was the task-relevant stimulus color categories. Previously, Ikeda and Osaka [18] found that it was likely that the *between- and within-category* colors were represented verbally by the phonological loop and visually by the visuospatial sketchpad, respectively. The findings of Experiment 1 agreed with the view. Experiment 2 expanded the findings to a shape-category task by showing that the Simon effect was eliminated in the *within-category* task and was generated in the *between-category* task, which indicated that the Simon effect was independent on the task-relevant stimulus attributes. Thus, Experiments 1 and 2 convergently demonstrate that the verbal WM representation is a critical factor for the generation of Simon effect.

Specially, Experiment 3 manifests that the task order is a critical modulation of the set effect [11]. In Experiments 3A and 3B, subjects adopted different WM encoding strategy to perform the *within-category* task. When the *between-category* task was run first (Experiment 3A), since subjects could acquire the verbal WM representation of the task-relevant stimulus attribute from the previous task (set effect), such a representation was adopted to perform the subsequent *within-category* task. Meanwhile, the spatial attribute of the stimulus was also represented, which interfered with the representation of color attribute, and therefore resulted in the Simon effect in the *within-category* task. Nevertheless, when the *within-category* task was performed first (Experiment 3B), there was no such a set effect. So, subjects performed the *within-category* task by representing the *within-category* colors in visual WM, which eliminated the Simon effect. In addition, the subsequent performance of the *between-category* condition was largely unaffected by the set effect since it was easily performed in the first place.

Notably, in the present study, the *between-* and *within-category* colors or shapes were used as the Simon stimuli, which exclusively resulted in that the *within-category* task-relevant stimuli were represented in visual WM system and the *between-category* task-relevant stimuli were represented in verbal WM system. However, one may raise a question that subjects may just make responses based on whether the color (or shape) is the same or different relative to the previous trial. However, if this is the case, the mean RTs will not be longer in the *within-category* task than those in the *between-category* task. Because adopting the mentioned response strategy will facilitate the stimulus processing. Moreover, the view that the *between- and within-category* stimuli are represented by the different WM codes had been strongly supported by the fMRI study [19], where Rothmayr et al. used the Gabor stimuli and found hemispheric dissociation in representing the verbal information by the phonological loop and the non-verbal information by visuospatial sketchpad.

Seemingly, the difference in task difficulty of the *within- and between-category* conditions may contaminate the present results, which was embodied in longer RTs in the *within-category* relative to *between-category* tasks because the former task was more difficult for subjects to perform. Previously, Hommel [32] deemed that the Simon effect would decay if the stimuli were more difficult to be discriminated. However, we think that whether the task is difficult or not is likely due to the adopted WM representation strategy when performing the *within- and between-category* tasks. Therefore, the internal reason of task difficulty likely derives from the difference in WM representation. That is, since visual WM representation strategy is adopted to perform the *within-category* task, the task is difficult, the RTs are lengthened, and the Simon effect declines; instead, since verbal WM representation strategy is adopted to perform the *within-category* task with the *between-category* task performed previously, the task becomes easier, the response are speeded up, and the Simon effect occurs.

In addition, studies [6], [26], [31], [32], [33] also found that the Simon effect reduced or even disappeared with prolonged RTs, which supports for the temporal activation account ascribing the Simon effect to the temporal overlap between the task-irrelevant response activation and the task-relevant response selection [26], [29], [32], [36]. That is, the task-irrelevant location-based response is activated quickly post-stimulus onset and then will rapidly decay away with the passage of time if the task-irrelevant response is incompatible with the task-relevant one. As a result, the task-irrelevant response activation interferes with the task-relevant response selection when the task-relevant stimulus attribute is quickly processed. In this case, there is large temporal overlap between the task-irrelevant response activation and the task-relevant response selection, resulting in the Simon effect. In contrast, if the processing of the task-relevant stimulus attribute is delayed due to the stimulus is more difficult to be discriminated, the temporal overlap between the task-irrelevant response activation and the task-relevant response selection will be smaller (or absent); in this case, the Simon effect will disappear.

In our Experiments 3A and 3B (Fig. 3), the results of the time course of the Simon effect as revealed by the RTs distribution analysis in both the *between- and within-category* tasks, which showed the reducing Simon effect with the lengthened RTs, seemed to support the account. However, we take this into consideration and offer another probable explanation. We argue that the verbal WM representation of task-relevant attribute is potential fundamental for the generation of the Simon effect because it reflects a flexible and efficient cognitive processing pattern that can be acquired according to both previous experience and current task requires. On one hand, when the task-relevant stimulus attribute is represented by verbal WM code, the processing of stimuli is effective and fast, so the RTs are short. In this case, the spatial dimension of the stimulus is expressed adequately. Since the processing of spatial dimension of the stimulus is more automatic [37], the spatial location information automatically interferes with the task-relevant response when they are incompatible, which results in the Simon effect. On the other hand, when the task-relevant stimulus attribute is represented by visual WM code, the processing of stimuli is hard and time-consuming. Probably, the visual WM representation of the *within-category* stimuli inhibits the representation of the spatial dimension of them, so RTs are overall long and the spatial effect cannot be expressed when the task-relevant and task-irrelevant responses are incompatible, which decreases the Simon effect.

Although the two possibilities, the temporal coding and the verbal WM representation of the task-relevant stimulus attribute, may explain why the Simon effect appears when the RTs are short, our account is also accordant with the existing evidences, which reveal that the Simon effect is related to the WM. In two influential studies [11], [12], Ansorge and Wühr designed the horizontal- and/or vertical-mapping Simon tasks and found that the Simon effect was only presented when the stimuli were presented on the spatial axis where subjects needed to discriminate, select, and represent the spatial codes. Zhao et al. [13] further investigated the influence of verbal and spatial WM loads on the Simon effect, and found that the Simon effect was eliminated by verbal WM load but did not be affected by spatial WM load. Some other researches [16], [38] also explored the generation of the Simon effect with a vocal response and found that the conceptual vocal response codes were activated during the Simon task, which therefore resulted in the reliable Simon effect. In addition, maybe some other interpretations can account for the present results; thus, more studies are needed to address the present issues.

## Conclusion

In three experiments, the present study finds some interesting results and extends previous work regarding the relationship between the generation of the Simon effect and the task-relevant attribute representations in WM system. The results of Experiments 1 and 2 indicate that the verbal WM representation is crucial for the generation of Simon effect. The results of Experiment 3 indicate that the set effect of the response-discrimination hypothesis depends on the task order and the reliable Simon effect occurs when subjects represented the task-relevant stimulus attribute by verbal WM encoding. Taken together, we conclude that the verbal WM representation can mediate the Simon effect although some other interpretations can account for the present results.
